# Differential Impact of Exercises on Quality-of-Life Improvement in Breast Cancer Survivors: A Network Meta-Analysis of Randomized Controlled Trials

**DOI:** 10.3390/cancers15133380

**Published:** 2023-06-28

**Authors:** Tzu-Chieh Wang, Pei-Lun Chen, Wan-Chun Liao, I-Chen Tsai

**Affiliations:** 1 Doctoral Program, Department of Nursing, College of Nursing, National Yang Ming Chiao Tung University, Taipei 112304, Taiwan; 48810251@yahoo.com.tw; 2 Department of Nursing, Taichung Veterans General Hospital, Taichung 407219, Taiwan; 3 InnovaRad, Taichung 407217, Taiwan; 4 Institute of Clinical Medicine, National Yang Ming Chiao Tung University, Taipei 112304, Taiwan; 5 Congenital Heart Disease Study Group, Asian Society of Cardiovascular Imaging, Seoul 13572, Republic of Korea

**Keywords:** breast cancer survivors, exercise interventions, quality of life, randomized controlled trials, aerobic and strength training

## Abstract

**Simple Summary:**

This study aims to find out which types of exercise can help improve the quality of life for people who have survived breast cancer. Researchers analyzed data from different studies to see how various exercises, such as aerobic and strength training, aerobic activity, yoga, and strength exercise, affected these individuals after 12 weeks. The results show that combining aerobic and strength training is the most effective way to improve their quality of life without causing more people to drop out of the exercise programs compared to regular care. This research may help doctors and patients make better decisions about exercise plans for breast cancer survivors.

**Abstract:**

This study aimed to assess the effectiveness of various exercise interventions in enhancing the quality of life for breast cancer survivors. To achieve this, randomized controlled trials were identified from major electronic databases, focusing on the relationship between exercise and quality of life in breast cancer survivors. The primary outcome was the impact of exercise on quality of life 12 weeks after the intervention, with a secondary outcome comparing dropout rates between intervention groups and a regular care control group. The study protocol was registered with INPLASY (INPLASY202340007). A network meta-analysis of nine randomized controlled trials involving 725 participants was conducted, examining aerobic and strength training, aerobic activity, yoga, and strength exercise. Results showed that aerobic and strength training was the most effective intervention, significantly improving the quality of life of breast cancer survivors (1.31; 95% confidence interval: 0.49 to 2.12). Aerobic activity had a borderline effect (0.83; 0.03 to 1.63), while no exercise interventions were associated with an increased dropout risk compared to the control group (regular care). The study concluded that concurrent aerobic and strength training can improve breast cancer survivors’ quality of life after 12 weeks of intervention without increasing dropout risk compared to regular care.

## 1. Introduction

Breast cancer is the most common female malignancy worldwide and has the fifth highest mortality rate of all cancers [1]. Owing to progress in cancer screening and advancements in cancer treatments, the number of breast cancer survivors in the United States exceeds 3.8 million, and it is estimated to rise by more than 30% in the next ten years [2]. Even after completing treatment, long-lasting and severe treatment-related side effects, such as physical problems, psychological distress, and impaired social and work reintegration, can still cause a significant decline in the quality of life for breast cancer survivors. Therefore, evidence-based care to support this population is an important issue for overall social health [3].

Exercise has been demonstrated to provide a multitude of advantages for individuals who have survived breast cancer, including improvements in physical function [4], fatigue [5], depression [6], and overall quality of life [7]. Nonetheless, a vast array of physical activity options exists, including aerobic activity [8], strength training [9], yoga [10], and others [11]. Current meta-analyses provide evidence that physical activity in general positively impacts the quality of life, but they do not offer insights into the specific amount or kind of exercise needed [7]. As a result, we currently do not know what types of exercise are effective prescriptions for breast cancer survivors after completing treatment, nor how long the intervention should last in order to see an effect. Understanding which exercise interventions are most effective for improving quality of life in breast cancer survivors as well as the expected duration of intervention required to observe positive effects is critical for developing effective rehabilitation programs.

Network meta-analysis represents a statistical technique that enables the concurrent evaluation of numerous interventions, facilitating the identification of the most efficacious exercise approaches [12]. The research methodology involves first collecting and categorizing various common interventions or treatments. Then, a network model is constructed, which allows for comparisons between different interventions to rank their effects. When there are studies directly comparing interventions head-to-head, these are referred to as direct comparisons. In cases where direct head-to-head comparisons are lacking between different treatments, indirect comparisons are made through a common comparator. For example, let us consider a mathematics exam scenario where, on average, student A scores 10 points higher than student B, and student B scores 5 points higher than student C. These are direct comparisons. However, through indirect estimation, we can infer that student A would likely score approximately 15 points higher than student C. This is known as an indirect comparison. To ensure the reliability of these indirect comparisons, network meta-analysis examines whether there are statistically significant differences between comparisons that have both direct and indirect evidence in order to establish internal consistency [12,13]. By choosing research conducted within a particular time range, it is possible to predict the exercise interventions that may yield statistically significant outcomes after a specific period of implementation. The objective of this network meta-analysis is to establish a hierarchy of the efficacy of various exercise interventions in enhancing the quality of life for breast cancer survivors and estimating the necessary time frame for observing statistically significant results. Such insights can assist in determining the most suitable exercise approach for breast cancer survivors aiming to enhance their overall well-being.

## 2. Materials and Methods

We conducted this study in accordance with the Preferred Reporting Items for Systematic Reviews and Meta-Analysis (PRISMA) extension guidelines for network meta-analysis (PRISMA NMA) [14]. The study was registered in INPLASY with the registration number INPLASY202340007 [15], and the ethical review board approval or participant informed consent was not required.

### 2.1. Database Searches and Study Identification

Two authors (TCW and ICT) performed separate electronic searches in PubMed, Cochrane Reviews, Cochrane CENTRAL, Web of Science, and ClinicalTrials.gov databases using the following keywords: (‘breast cancer’) AND (‘quality of life’ OR ‘QoL’) AND (‘exercises’ OR ‘physical activity’ OR ‘yoga’ OR ‘aerobic’) AND (‘random’ OR ‘randomized’ OR ‘randomised’) AND (‘12 weeks’ OR ‘3 months’). The search approach for the systematic review and network meta-analysis spanned the duration from the first available entry in each database up to the most recent search date (7 April 2023).

In the initial stage, two authors were tasked with evaluating the titles and abstracts of identified studies for their eligibility using a consensus process. The search was conducted in the aforementioned databases to scrutinize eligible trials. Additionally, the reference lists of various review articles [5,7,16,17,18,19,20,21,22,23,24] were examined and manual searches were performed. In situations where the two initial reviewers were unable to reach a consensus, a third reviewer and study author (PLC) was consulted. No restrictions on language were imposed on this search.

### 2.2. Inclusion and Exclusion Criteria

The network meta-analysis employed the PICO model (population, intervention, comparison, outcome), featuring the subsequent criteria: (1) P: human participants with breast cancer and completed treatment, including surgery, chemotherapy, and/or radiotherapy; (2) I: exercise interventions; (3) C: control group without intervention; and (4) O: changes in quality of life. The definition of breast cancer survivor was based on the joint guideline provided by the American Cancer Society and the American Society of Clinical Oncology [25].

The study applied the following inclusion criteria: (1) randomized controlled trials that recruited breast cancer survivors who had completed treatments, including surgery, chemotherapy, and/or radiation therapy, (2) randomized controlled trials that investigated the quantitative assessment of quality of life after exercise intervention, (3) the control group that received no intervention or regular care, and (4) trials that had available data on quality of life pre- and post-intervention at 12 weeks.

The selection of the 12-week evaluation duration was based on the initial literature review, which indicated that it was the most commonly used assessment period in the included studies. Several previous large-scale literature analyses have also found that the onset of exercise effects occurred at 12 weeks for patients undergoing rehabilitation [26] after stroke or a transient ischemic attack [27]. In order to compare the effectiveness of various exercise interventions, a standardized time frame is necessary to establish a benchmark for comparison. Therefore, this study focuses specifically on a 12-week duration and excludes other time frames with fewer studies available.

Exclusion criteria for this review and network meta-analysis included: (1) non-randomized controlled trials, (2) studies without comparisons of exercise vs. exercise or exercise vs. regular care comparison, (3) studies lacking quantitative assessments of quality of life, (4) studies quantitatively assessed quality of life but only reported subscale data and did not provide a total score, (5) incomplete or unavailable data, even after attempts to contact the authors via email, and (6) studies enrolling participants overlapped with a published trial already enrolled in our analysis.

### 2.3. Modeling for Network Meta-Analysis

In the present network meta-analysis, we adhered to the following principles during the construction of the model. To prevent excessive heterogeneity, we restricted the paired comparisons to only exercise vs. exercise or exercise vs. regular care. Comparisons between exercise and cognitive behavioral therapy, eurythmy therapy, and various nutritional supplements were thus excluded. Inclusion of additional treatments might result in disparate network geometries, owing to the variation in the therapies being considered, leading to inconsistent outcomes in the network meta-analysis [28].

When categorizing the exercise type in our study, they were grouped based on the actual exercise prescription content discussion between two authors (TCW, ICT). If there is any disagreement in the categorization, consensus will be reached through discussion with the third author (PLC).

### 2.4. Methodological Quality Appraisal

To assess the methodological quality of the studies included in our analysis, we utilized the Cochrane risk of bias tool for randomized trials (version 2, RoB 2, London, UK) [29]. This tool appraises six principal components for assessing the quality of a study, including the randomization process, adherence to the intervention, missing outcome data, outcome measurement, selective reporting, and overall risk of bias.

### 2.5. Primary Outcome: Quality-of-Life Improvement, Standardized Mean Difference

The primary outcomes evaluated in this study were changes in quality of life measured by quantitative scales. If the study utilized a breast-cancer-specific quality-of-life scale, such as the Functional Assessment of Cancer Therapy-Breast [30,31] or the International Breast Cancer Study Group Quality of Life [32], data extraction was prioritized from these scales. If the study did not use a breast-cancer-specific quality-of-life scale, data extraction was prioritized in the following order: cancer-specific quality-of-life assessment tools, such as the European Organization for Research and Treatment of Cancer Quality-of-Life Questionnaire [33], followed by general quality-of-life assessment tools, such as the Functional Assessment of Cancer Therapy-General [34].

### 2.6. Secondary Outcome: Risk Difference of Dropout Rates

The secondary outcome measure was the risk difference of dropout rates at the 12th week, which provides an intuitive indicator. For example, if an individual chooses a specific exercise regimen to improve their quality of life and experiences a dropout rate of 12%, while the control group, which only receives regular care, has a dropout rate of 7% (which may result in some of them starting an exercise routine on their own), the risk difference in dropout rates would be 5%.

### 2.7. Data Extraction, Management and Conversion

Two authors (TCW and ICT) performed the data extraction process independently, including demographic information, study design, exercise protocol details, and primary and secondary outcomes from the evaluated studies. In situations where the necessary data were not available in the published articles, we reached out to the corresponding authors to obtain the primary data.

Data extraction, conversion, and result merging were conducted in accordance with the recommendations outlined in the Cochrane Handbook for Systematic Reviews of Interventions and relevant medical literature [12,35,36,37,38].

### 2.8. Statistical Analyses

Due to the inclusion of various exercise types, a random-effects model was utilized for the network meta-analysis [39]. The analysis was performed using MetaInsight (version 4.0.2, Complex Reviews Support Unit, National Institute for Health Research, London, UK) under a frequentist framework. MetaInsight represents a web-based platform for network meta-analysis that leverages the *netmeta* package in R software for conducting frequentist statistical calculations [40].

Initially, a forest plot and network plot were generated to display all pairwise comparisons from individual studies. Subsequently, forest plots were created for standardized mean differences in the change of quality of life at 12 weeks and the risk differences of dropout rates for each exercise type compared to the control group to provide an overall summary of the effects [41]. The effect sizes were presented as point estimates with a 95% confidence interval (95% CI) [41]. The exercise types were ranked, and numerical values for both direct and indirect comparisons were presented in tables. Inconsistency tests were conducted to detect any data disparities. Statistical significance was defined as a two-tailed *p* value of less than 0.05.

### 2.9. Sensitivity Analyses

Two sensitivity analyses were conducted to strengthen the robustness of the study findings. The first analysis employed a one-study removal method, which was performed to ensure that the effect estimates of individual studies did not excessively influence the overall results. Sequentially removing one study at a time from the analysis of quality-of-life changes at 12 weeks allowed us to determine whether the study conclusions and ranking remained consistent.

The second sensitivity analysis performed in this study involved the pre-post correlation coefficient. When transforming baseline and post-intervention quality-of-life measurements into mean and standard deviation of changes, it is necessary to assume a pre–post correlation coefficient. In this study, a coefficient of 0.8 was utilized, as recommended by the Cochrane handbook [35]. However, different scholars may hold varying opinions on this coefficient with commonly used values being 0.5, 0.7, and 0.8 [42]. To examine whether the selected coefficient would impact the study results, a sensitivity analysis was conducted by calculating the effect sizes of quality-of-life changes at 12 weeks with a coefficient of 0.5 [42]. The direction, size of the effect, statistical significance, and ranking of the results were assessed.

### 2.10. Publication Bias

Potential publication bias was assessed in accordance with the Cochrane Handbook for Systematic Reviews of Interventions [12]. The funnel plot was generated using Comprehensive Meta-Analysis software, version 4 (Biostat, Englewood, NJ, USA), based on the comparison with the control group. Additionally, an Egger’s regression test was conducted to quantify the presence of significant publication bias.

## 3. Results

### 3.1. Study Identification and Network Model Formation

**Figure 1:**
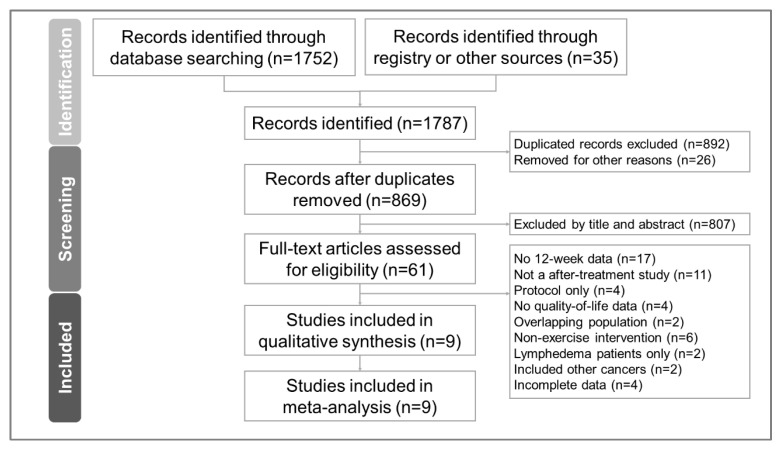
Flow diagram for the study selection process based on the Preferred Reporting Items for Systematic Reviews and Meta-Analyses (PRISMA) guidelines.

The PRISMA flowchart detailing the literature search is presented in Figure 1. The PRISMA NMA extension’s checklist is provided in Table S1. The number of articles retrieved from various databases is presented in Table S2. After removing duplicate articles and excluding non-relevant articles by screening titles and abstracts, we ultimately included nine randomized controlled trials [6,8,9,10,11,43,44,45,46]. The articles excluded in the final stage [4,47,48,49,50,51,52,53,54,55,56,57,58,59,60,61,62,63,64,65,66,67,68,69,70,71,72,73,74,75,76,77,78,79,80,81,82,83,84,85,86,87,88,89,90,91,92,93,94,95,96,97] along with their respective reasons for exclusion are listed in Table S3.

**Figure 2:**
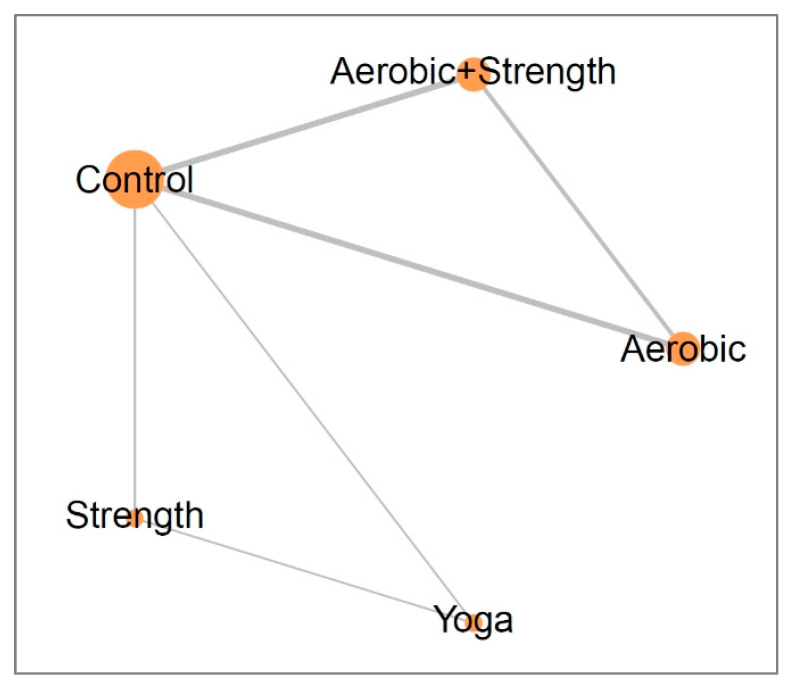
Network plots illustrate the effects of different exercise interventions on the improvement of quality of life in breast cancer survivors after 12 weeks. The size of each node and thickness of each line represents the number of trials included in the analysis.

Our analysis included a total of nine randomized controlled trials, involving 725 individuals. Based on the included studies, the exercise types were categorized as follows: aerobic and strength training (concurrent), aerobic activity, yoga, and strength exercise. The network model for the exercise interventions is displayed in Figure 2.

Among the nine studies included in our analysis, three studies exclusively recruited postmenopausal women [6,10,44], and two studies only enrolled patients with fatigue [11,45]. For further details on the inclusion criteria, the country where the study was conducted, the mean age and standard deviation of the participants, exercise intervention details, quality-of-life assessment scales, and dropout rates, please refer to Table 1.

### 3.2. Methodological Quality of the Included Studies

Regarding the overall methodological quality of the studies, we observed that 44.4% (4/9) of the studies had a low risk of bias, while 55.6% (5/9) had some risk of bias (refer to Figure S1). The studies with some risk of bias had differences in their protocols between study arms, which could potentially impact the adherence and outcomes of the interventions. The details of the risk of bias assessment are provided in Table S4.

### 3.3. Primary Outcome: Aerobic and Strength Concurrent Training Most Effective

**Figure 3:**
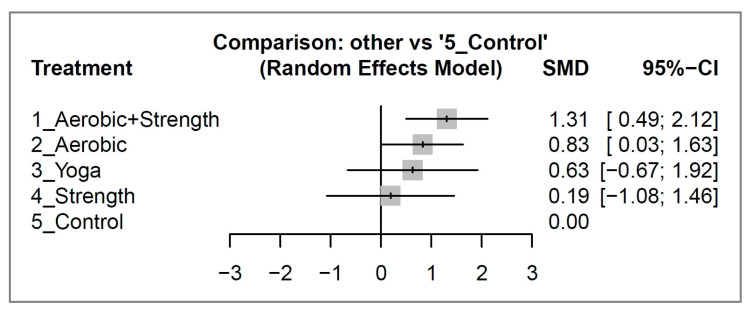
Forest plots illustrating the standardized mean difference (SMD) in quality-of-life improvement between different exercise interventions and control groups among breast cancer survivors after 12 weeks of intervention.

After a 12-week intervention, aerobic and strength training showed a significant improvement in quality of life (effect size: 1.31; 95% CI: 0.49 to 2.12), while aerobic activity demonstrated a borderline effect (effect size: 0.83; 95% CI: 0.03 to 1.63). On the other hand, yoga (effect size: 0.63; 95% CI: −0.67 to 1.92) and strength training (effect size: 0.19; 95% CI: −1.08 to 1.46) did not show a significant difference compared to the control group (Figure 3). Please refer to Figure S2 for the detailed pair-wise comparisons between study arms as reported in individual studies.

The exercise interventions were ranked based on their effect sizes on quality of life, with aerobic and strength training (concurrent) being the most effective, followed by aerobic activity, yoga, and strength exercise in that order. Please see Table 2 for a detailed comparison and ranking of the exercise types.

### 3.4. Secondary Outcome: Dropout Rates Statistically Similar

**Figure 4:**
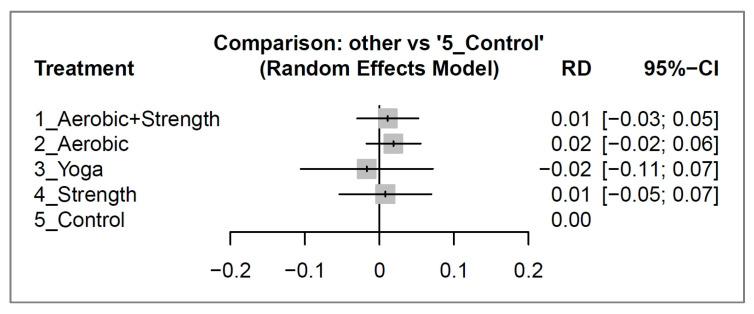
Forest plots depicting the risk difference (RD) of dropout rates between different exercise interventions and control groups for breast cancer survivors after 12 weeks of intervention.

After 12 weeks of intervention, there was no significant difference in dropout rates between the various exercise types and the control group with all risk differences with their 95% CIs overlapped with 0 (see Figure 4). For a detailed analysis of the pair-wise comparisons between study arms as reported in individual studies, please consult Figure S3.

### 3.5. Inconsistency Test

The network was constructed by creating nodes and performing direct and indirect comparisons to determine consistency. The results of the quality-of-life inconsistency tests are presented in Table S5, while the dropout rate results are presented in Table S6. All available comparisons had *p* values greater than 0.05, indicating no evidence of inconsistency between direct and indirect comparisons.

### 3.6. Sensitivity Analyses

The results of the one-study removal analysis showed consistent rankings and clinical significance for all exercise types. The aerobic and strength-training intervention consistently demonstrated a significant improvement in the quality of life of breast cancer survivors, while the aerobic activity intervention remained at borderline significance. Yoga and strength exercise interventions consistently showed no significant effect on quality of life (See Figure S4a–i).

In the second sensitivity analysis, we adjusted the pre–post correlation coefficient from 0.8 to 0.5 and conducted a new network comparison (Figure S5). Our results showed that the direction of effect sizes, ranking, and interpretation of the results remained consistent with those obtained using a coefficient of 0.8 (Figure 3).

The above analyses indicate that the results of our study are consistent and not influenced by the inclusion or removal of individual studies as well as the adjustment of assumed values in the calculation process.

### 3.7. Publication Bias

Please see Figure S6 for the funnel plot. The Egger’s test yielded a *p* value of 0.25, indicating no significant publication bias.

## 4. Discussion

### 4.1. Main Findings and Clinical Implications

Our network meta-analysis revealed that among breast cancer survivors, aerobic and strength training was the most effective type of 12-week exercise intervention in improving quality of life (effect size: 1.31; 95% CI: 0.49 to 2.12). Aerobic activity had a borderline effect (effect size: 0.83; 95% CI: 0.03 to 1.63), while yoga and strength exercise showed no significant difference compared to the control group. In terms of dropout rates, there was no significant risk difference between the different types of exercise and the control group. For breast cancer survivors and caregivers, our network meta-analysis provides valuable information for exercise prescription. The data can be used to support the benefits of exercise and encourage patients to adhere to the exercise program for at least three months to achieve a significant improvement in quality of life.

### 4.2. Significance of the Findings Compared to Existing Literature

Aune et al. published a comprehensive pairwise meta-analysis in *JNCI Cancer Spectrum* in 2022 [7], which collected 79 randomized controlled trials and 14,554 breast cancer patients before 2019, including various exercise protocols and intervention durations. The study concluded that physical activity, compared to regular care, can effectively improve global health-related quality of life. However, the authors also stated that based on their analysis, the evidence regarding the dose and type of physical activity is still insufficient to draw conclusions.

Our study utilized network meta-analysis to compare various exercise interventions and concluded that within a 12-week timeframe, (concurrent) aerobic and strength training is the most effective type of exercise for improving quality of life in breast cancer survivors, followed by aerobic activity with a borderline effect. This study is the first in the literature to provide answers to questions regarding the effectiveness of different types of exercise, their comparison, and the ranking of exercise benefits.

Previously, studies often mentioned that yoga is beneficial for breast cancer survivors [98,99]. However, some of these studies relied on self-reported surveys and lacked prospective designs with specific intervention durations. They included patients with different frequencies and durations of yoga interventions [98]. Some systematic reviews also incorporated breast cancer patients during and after treatment without specifying the exact duration of yoga intervention [99]. In our study, we directly used a 12-week timeframe as the research benchmark and compared and ranked the effects of yoga on quality of life among various exercises. In other words, we are not answering whether yoga is effective for breast cancer survivors, but rather, within the 12-week timeframe, we assessed the varying impact on quality of life from different exercises performed by breast cancer survivors with yoga being part of the ranking results.

### 4.3. Possible Explanations for the Observed Results

Regarding the ranking of the effectiveness of different types of exercise in improving quality of life, we hypothesize that the intensity of the exercise may play a role. Ostman et al. found that the improvement in quality of life is more pronounced with increasing exercise intensity in patients with heart failure [100]. In the exercise protocols designed for breast cancer survivors in our included studies [6,8,11,43,44,46], aerobic exercise is easier to perform, can be sustained for longer durations, and is more likely to achieve moderate or even vigorous intensity. This may suggest that exercise interventions incorporating aerobic activity, such as concurrent aerobic and strength training and aerobic activity only, tend to result in better outcomes. In Dysart et al.’s study, yoga has been found to achieve moderate intensity only 32.75% of the time on average, and most of the time, it only achieves low intensity [101]. As for the strength-exercise-only protocols, our included studies consisted of a home-based exercise program without additional weight bearing [45] and a program based on 40% of one repetition maximum (1RM), gradually increasing to 70% 1RM based on the participant’s capacity with the help of a professional trainer [9]. Day et al.’s previous research on the correspondence between resistance training and exercise intensity suggests that 40%, 70%, and 90% 1RM correspond to low, moderate, and vigorous intensity, respectively [102]. Thus, a 40–70% 1RM training protocol [9] corresponds only to low-to-moderate intensity. Moreover, even at 70% 1RM, the actual exercise time of 12 lifts is shorter than that of aerobic exercise.

The lack of significant differences in dropout rates between the exercise interventions and regular care may be attributed to the design of the exercise protocols, which were easily followed. For instance, the yoga classes were led by professional instructors and provided a social component, lasting for 60–90 min [10,45]. The strength-training intervention was facilitated by professional trainers and included progressive overload, leading to a sense of accomplishment after each session [9]. Even the self-administered aerobic activities were completed within an hour, preventing excessive difficulty [44].

### 4.4. Limitations

Our study has limitations. Among the included studies, three studies enrolled only postmenopausal women and two studies enrolled breast cancer survivors with fatigue, which may violate the transitivity assumption due to the heterogeneous study population. However, based on the age distribution of the included participants, it was noted that the age range of participants in studies without specific menopause inclusion criteria was mostly in the postmenopausal phase (Table 1). Additionally, a previous study conducted by Álvarez-Bustos et al. investigated the prevalence of fatigue in breast cancer survivors and reported that only 9% of participants reported no fatigue at all [103]. These findings suggest that the actual participants included in these nine studies were not significantly different from each other, which supports the assumption of transitivity in network meta-analysis. As a confirmation, our study passed the inconsistency test and the sensitivity analysis of one-study removal, indicating that no specific study or study group caused inconsistency or instability in the results.

Furthermore, our study only investigated the effect of 12 weeks of exercise on quality of life, and it is unknown whether exercise types that did not show significant effects at 12 weeks might lead to improvements with longer duration of exercise (e.g., 24 or 48 weeks). Future network meta-analyses with longer follow-up periods are needed to investigate this question. However, we found that studies with longer intervention periods, such as 24-week ones [55,56,57,58,59,60,61], were less abundant in our literature review, and their results may not be directly comparable or applicable to our 12-week study.

## 5. Conclusions

In summary, for breast cancer survivors, aerobic and strength concurrent training for 12 weeks is the exercise of choice to improve quality of life, with dropout rates comparable to the control group.

## Figures and Tables

**Table 1:** Summary of the included trials investigating the effect of exercise to improve quality of life in breast cancer survivors.

First Author & Year	Study Country	Enrolled Population (Age ^1^)	Participants in Nodes	QoL Scale (Range)	12-Week QoL Improvement	12-Week Dropouts	Exercise Details
Milne 2008 [43]	Australia	Stage I–II breast cancer completed all treatments except H/T (55.1 ± 8.2)	Aerobic + Strength 29 Control 29	FACT-B (0–144)	20.80 ± 7.80 −5.30 ± 8.63	0/29 0/29	The intervention consisted of 20 min of aerobic activity with a 5-min cool down, followed by 12 strength-training movements, each performed for 2 sets of 10–15 repetitions. The intervention was conducted 3 times a week for 12 weeks.
Ergun 2013 [6]	Turkey	Stage I–III breast cancer s/p op, C/T, R/T in post-menopause (51.7 ± 8.8)	Aerobic + Strength 20 Aerobic 18 Control 20	EORTC QLQ-C30 (0–100)	6.25 ± 11.33 7.73 ± 14.21 −6.67 ± 14.24	0/20 2/20 0/20	Group 1: Strength + Aerobic intervention, which included resistance training for 45 min per day, 3 days per week, and brisk walking for 30 min per day, 3 days per week. Group 2: Aerobic intervention, which included brisk walking for 30 min per day, 3 days per week. Group 3: Control group, which received no specific intervention.
Baruth 2015 [44]	USA	Stage I–III breast cancer completed adjuvant treatment in post-menopause (56.5 ± 6.3)	Aerobic 18 Control 12	IBCSG QOL (0–100)	5.50 ± 15.81 −3.90 ± 15.42	2/20 0/12	Participants engaged in instructed walking as the intervention, starting from 20 min per day, 3 days per week with an RPE of 3 (on a scale of 0–10), and gradually increasing to 30–40 min per day, 5 days per week with an RPE of 4–6 over the 12-week period.
Cramer 2015 [10]	Germany	Stage I–III breast cancer s/p op, C/T, R/T in post-menopause (49.2 ± 5.9)	Yoga 19 Control 21	FACT-B (0–144)	10.80 ± 12.87 −1.90 ± 8.89	0/19 0/21	Participants engaged in a Hatha yoga intervention led by a certified instructor for 90 min once per week over a 12-week period.
Rogers 2015 [8]	USA	DCIS or Stage I-IIIa breast cancer s/p op, C/T, R/T (54.4 ± 8.5)	Aerobic 106 Control 110	FACT-B (0–144)	5.10 ± 11.08 −0.60 ± 12.70	4/110 2/112	The intervention involved gradually increasing aerobic exercise over 12 weeks, starting with 15–20 min, 3 days a week at 40–59% of heart rate reserve and progressing to moderate intensity (>3 times per week, 30–50 min, 40–59% heart rate reserve).
Stan 2016 [45]	USA	Stage 0–II breast cancer s/p op, C/T, R/T with cancer-related fatigue (62.1 ± 8.1)	Yoga 14 Strength 9	FACT-B (0–144)	5.50 ± 9.70 7.00 ± 10.70	4/18 7/16	Yoga intervention: Participants engaged in a 90-min video program, 3–5 times per week. Strength intervention: Participants engaged in 5 upper and 5 lower body exercises with 8–10 repetitions per exercise, for a total of 20 min, 3–5 times per week.
Kim 2020 [11]	Korea	Stage I–III breast cancer completed op and C/T with fatigue (49.2 ± 7.1)	Aerobic + Strength 23 Control 25	FACT-B (0–144)	32.85 ± 15.10 28.40 ± 16.10	1/24 1/26	The 12-week comprehensive program included social group interaction and a combination of low-, moderate-, and high-intensity exercises, consisting of both aerobic and strength training. Participants received three exercise sessions per week.
Soriano-Maldonado 2022 [9]	Spain	Non-metastatic breast cancer s/p op, C/T, R/T (52.3 ± 9.0)	Strength 32 Control 28	FACT-B + 4 (0–148)	0.00 ± 9.62 2.90 ± 9.52	0/32 0/28	Participants engaged in a resistance-training intervention led by an exercise professional, which consisted of 60 min per session, twice per week, for a total of 12 weeks. The resistance training was initiated with a weight load corresponding to 40% of the participants’ 1RM and was gradually increased to 70% of their 1RM weight based on their ability to tolerate the load.
Lin 2023 [46]	China	Breast cancer s/p op (51.6 ± 30.7)	Aerobic 145 Aerobic + Strength 47	FACT-B (0–144)	7.12 ± 11.72 12.19 ± 12.28	5/150 3/50	Group 0: JME (a 15-min exercise at 60–80% of HRmax, 3 times a day). Group 1: JME with follow-up. Group 2: JME with aerobic activity (30 min, 5 times per week). Groups 0, 1, and 2 were combined as the aerobic exercise intervention. Group 3: JME with resistance training (8 movements with progressive loads, 2–3 times per week). Group 3 was categorized as the aerobic + strength intervention.

^1^ The mean age along with its standard deviation is reported in years as the unit of measurement. 1RM: one repetition maximum; C/T: chemotherapy; DCIS: ductal carcinoma in situ; EORTC QLQ-C30: European Organization for Research and Treatment of Cancer Quality-of-Life Questionnaire; FACT-B: Functional Assessment of Cancer Therapy-Breast; FACT-B + 4: Functional Assessment of Cancer Therapy-Breast with Lymphedema; HRmax: maximum heart rate; H/T: hormonal therapy; IBCSG QOL: International Breast Cancer Study Group Quality of Life; JME: joint motility exercise; op: operation (surgery); QoL: quality of life; RPE: rating of perceived exertion; R/T: radiotherapy; s/p: status post; USA: United States of America.

**Table 2:** Pairwise comparison and ranking of different exercise interventions for improving quality of life at 12 weeks in breast cancer survivors.

**Aerobic + Strength**	0.18 [−0.90, 1.25]	-	-	1.42 [0.51, 2.33]
0.48 [−0.40, 1.36]	**Aerobic**	-	-	0.71 [−0.18, 1.60]
0.68 [−0.85, 2.21]	0.200 [−1.32, 1.72]	**Yoga**	−0.15 [−1.81, 1.51]	1.16 [−0.42, 2.74]
1.12 [−0.39, 2.62]	0.64 [−0.86, 2.14]	0.44 [−0.89, 1.76]	**Strength**	−0.30 [−1.82, 1.22]
1.31 [0.49, 2.12]	0.83 [0.03, 1.63]	0.63 [−0.67, 1.92]	0.19 [−1.08, 1.46]	**Control**

The estimates from pairwise meta-analyses are located above the diagonal line, while the estimates from network meta-analyses are located below the diagonal line.

## Data Availability

Data are contained within the article and Supplementary Files.

## Supplementary Materials

The following supporting information can be downloaded at: https://www.mdpi.com/article/10.3390/cancers15133380/s1, Table S1: PRISMA for network meta-analysis checklist; Table S2: Keywords and search results in different databases; Table S3: Excluded studies and reasons; Table S4: Detailed quality assessment of included studies; Table S5: Inconsistency test results of quality of life improvement for breast cancer survivors; Table S6: Inconsistency test results for risk difference of dropout rates; Figure S1: Summary of quality assessment for the studies included; Figure S2: The forest plot of pair-wise comparisons for quality of life improvement; Figure S3: The forest plot of pair-wise comparisons for the risk difference of dropout rates; Figure S4: Sensitivity analysis with the one-study removal method; Figure S5: Sensitivity analysis with pre-post correlation coefficient changed from 0.8 to 0.5; Figure S6: Funnel plot of all paired comparisons involving the common comparator, control group.
