# Supplementary material for: Differential Impact of Exercises on Quality-of-Life Improvement in Breast Cancer Survivors: A Network Meta-Analysis of Randomized Controlled Trials
Source: Cancers (Basel). 2023 Jun 28;15(13):3380. doi: 10.3390/cancers15133380 (PMC10341084; doi:10.3390/cancers15133380)
Supplement: Supplementary file 1 [file cancers-15-03380-s001.zip › cancers-2401033-supplementary.pdf]

Supplementary Tables and Figures

Table S1 - PRISMA for network meta-analysis checklist

Section and Topic	#	Checklist item	Location
**Title**			
Title	1	Identify the report as a systematic review incorporating a network meta-analysis (or related form of meta-analysis).	Title
**Abstract**			
Structured summary	2	Provide a structured summary including, as applicable: **Objective**: main objectives / **Methods**: data sources; study eligibility criteria, participants, and interventions; study appraisal; and synthesis methods, such as network meta-analysis. / **Results**: number of studies and participants identified; summary estimates with corresponding confidence/credible intervals; treatment rankings may also be discussed. Authors may choose to summarize pairwise comparisons against a chosen treatment included in their analyses for brevity. / **Conclusions**: limitations; conclusions and implications of findings. / **Other**: primary source of funding; systematic review registration number with registry name.	Abstract
**Introduction**			
Rationale	3	Describe the rationale for the review in the context of what is already known, including mention of why a network meta-analysis has been conducted.	Introduction / 1st and 2nd paragraph
Objectives	4	Provide an explicit statement of questions being addressed, with reference to participants, interventions, comparisons, outcomes, and study design (PICOS).	Introduction / 3rd paragraph
**Methods**			
Protocol & registration	5	Indicate whether a review protocol exists and where it can be accessed; and, if available, provide registration information, including registration number.	Materials and Methods / 1st paragraph
Eligibility criteria	6	Specify study characteristics (e.g., PICOS, length of follow-up) and report characteristics (e.g., years considered, language, publication status) used as criteria for eligibility, giving rationale. Clearly describe eligible treatments included in the treatment network, and note whether any have been clustered or merged into the same node (with justification).	Materials and Methods / Inclusion and exclusion criteria
Information sources	7	Describe all information sources (e.g., databases with dates of coverage, contact with study authors) in the search and date last searched.	Table S2
Search	8	Present full electronic search strategy for at least one database, including any limits used, such that it could be repeated.	Table S2
Study selection	9	State the process for selecting studies (i.e., screening, eligibility, included in systematic review, and, if applicable, included in the meta-analysis).	Materials and Methods / Study identification / Inclusion and exclusion criteria
Data collection	10	Describe method of data extraction from reports (e.g., piloted forms, independently, in duplicate) and any processes for obtaining and confirming data.	Materials and Methods / Data extraction
Data items	11	List and define all variables for which data were sought (e.g., PICOS, funding sources) and any assumptions and simplifications made.	Materials and Methods / Data extraction and conversion
Network geometry	S1	Describe methods used to explore the geometry of the treatment network under study and potential biases related to it. This should include how the evidence base has been graphically summarized for presentation, and what characteristics were compiled and used to describe the evidence base to readers.	Materials and Methods / Modeling for network meta-analysis
Risk of bias within	12	Describe methods used for assessing risk of bias of individual studies (including specification of whether this was done at the study or outcome level), and how this information is to be used in any data synthesis.	Materials and Methods / Quality appraisal
Summary measures	13	State the principal summary measures (e.g., risk ratio, difference in means). Also describe the use of additional summary measures assessed, such as treatment rankings, as well as modified approaches used to present summary findings from meta-analyses.	Materials and Methods / Outcomes

Planned methods of analysis	14	Describe the methods of handling data and combining results of studies for each network meta-analysis. This should include, but not be limited to: Handling of multi-arm trials; Selection of variance structure; Selection of prior distributions in Bayesian analyses; and Assessment of model fit.	Materials and Methods / Statistical analyses
Assessment of inconsistency	S2	Describe the statistical methods used to evaluate the agreement of direct and indirect evidence in the treatment network(s) studied. Describe efforts taken to address its presence when found.	Materials and Methods / Statistical analyses
Risk of bias across	15	Specify any assessment of risk of bias that may affect the cumulative evidence.	Materials and Methods / Publication bias
Additional analyses	16	Describe methods of additional analyses if done, indicating which were pre-specified. This may include, but not be limited to, the following: Sensitivity or subgroup analyses; Meta-regression analyses; Alternative formulations of the treatment network; and Use of alternative prior distributions for Bayesian analyses (if applicable).	Materials and Methods / Sensitivity analyses
**Results**			
Study selection	17	Give numbers of studies screened, assessed for eligibility, and included in the review, with reasons for exclusions at each stage, ideally with a flow diagram.	Results / Study identification Figure 1, Table S2, Table S3
Network structure	S3	Provide a network graph of the included studies to enable visualization of the geometry of the treatment network.	Figure 2
Network geometry	S4	Provide a brief overview of characteristics of the treatment network. This may include commentary on the abundance of trials and randomized patients for the different interventions and pairwise comparisons in the network, gaps of evidence in the treatment network, and potential biases reflected by the network structure.	Results / Network model formation / Figure 2
Study characteristics	18	For each study, present characteristics for which data were extracted (e.g., study size, PICOS, follow-up period) and provide the citations.	Table 1
Risk of bias within	19	Present data on risk of bias of each study and, if available, any outcome level assessment.	Table S4, Figure S1, Methodological quality
Results of individual studies	20	For all outcomes considered (benefits or harms), present, for each study: (1) simple summary data for each intervention group, and (2) effect estimates and confidence intervals. Modified approaches may be needed to deal with information from larger networks.	Table 1
Synthesis of results	21	Present results of each meta-analysis done, including confidence/credible intervals. In larger networks, authors may focus on comparisons versus a particular comparator (e.g. placebo or standard care), with full findings presented in an appendix. League tables and forest plots may be considered to summarize pairwise comparisons. If additional summary measures were explored (such as treatment rankings), these should also be presented.	Outcomes / Figure 3, Figure 4, Figure S2, Figure S3, Table 2
Exploration for inconsistency	S5	Describe results from investigations of inconsistency. This may include such information as measures of model fit to compare consistency and inconsistency models, P values from statistical tests, or summary of inconsistency estimates from different parts of the treatment network.	Inconsistency test Table S5, Table S6
Risk of bias across	22	Present results of any assessment of risk of bias across studies for the evidence base being studied.	Publication bias, Figure S6
Additional analyses	23	Give results of additional analyses, if done (e.g., sensitivity or subgroup analyses, meta-regression analyses, alternative network geometries studied, alternative choice of prior distributions for Bayesian analyses, and so forth).	Sensitivity analysis / Figure S4, Figure S5
**Discussion**			
Summary of evidence	24	Summarize the main findings, including the strength of evidence for each main outcome; consider their relevance to key groups.	Discussion Findings and implications
Limitations	25	Discuss limitations at study and outcome level (e.g., risk of bias), and at review level (e.g., incomplete retrieval of identified research, reporting bias). Comment on the validity of the assumptions, such as transitivity and consistency. Comment on any concerns regarding network geometry (e.g., avoidance of certain comparisons).	Discussion Limitations
Conclusions	26	Provide a general interpretation of the results in the context of other evidence, and implications for future research.	Conclusion
**Funding**			
Funding	27	Describe sources of funding for the systematic review and other support (e.g., supply of data); role of funders for the systematic review. This should also include information regarding whether funding has been received from manufacturers of treatments in the network and/or whether some of the authors are content experts with professional conflicts of interest that could affect use of treatments in the network.	Funding

PICOS, population, intervention, comparators, outcomes, study design.

Table S2 - Keywords and search results in different databases

Database	Keyword	Filter	Date	Results
PubMed	('breast cancer') AND ('quality of life' OR 'QoL') AND ('exercises' OR 'physical activity' OR 'yoga' OR 'aerobic') AND ('random' OR 'randomized' OR 'randomised') AND ('12 weeks' OR '3 months)	NA	April 7, 2023	360
Cochrane Reviews	('breast cancer') AND ('quality of life' OR 'QoL') AND ('exercises' OR 'physical activity' OR 'yoga' OR 'aerobic') AND ('random' OR 'randomized' OR 'randomised') AND ('12 weeks' OR '3 months)	Title Abstract Keyword	April 7, 2023	14
Cochrane CENTRAL	('breast cancer') AND ('quality of life' OR 'QoL') AND ('exercises' OR 'physical activity' OR 'yoga' OR 'aerobic') AND ('random' OR 'randomized' OR 'randomised') AND ('12 weeks' OR '3 months)	Title Abstract Keyword	April 7, 2023	769
Web of Science	('breast cancer') AND ('quality of life' OR 'QoL') AND ('exercises' OR 'physical activity' OR 'yoga' OR 'aerobic') AND ('random' OR 'randomized' OR 'randomised') AND ('12 weeks' OR '3 months)	NA	April 7, 2023	608
ClinicalTrials.gov	('breast cancer') AND ('quality of life' OR 'QoL') AND ('exercises' OR 'physical activity' OR 'yoga' OR 'aerobic') AND ('random' OR 'randomized' OR 'randomised') AND ('12 weeks' OR '3 months)	Condition or disease	April 7, 2023	1

NA: not applied

Table S3 - Excluded studies and reasons

Citations	Reasons
Heiman, J., Onerup, A., Bock, D., Haglind, E., Olofsson Bagge, R., 2022. The effect of nonsupervised physical activity before and after breast cancer surgery on quality of life: Results from a randomized controlled trial (PhysSURG-B). Scand J Surg 111 (4), 75-82.	No 12-week data. Only 4-week data.
Mostafaei, F., Azizi, M., Jalali, A., Salari, N., Abbasi, P., 2021. Effect of exercise on depression and fatigue in breast cancer women undergoing chemotherapy: A randomized controlled trial. Heliyon 7 (7), e07657.	No 12-week data. Only 6- and 10-week data.
Naczk, A., Huzarski, T., Doś, J., Górska-Doś, M., Gramza, P., Gajewska, E., Naczk, M., 2022. Impact of inertial training on muscle strength and quality of life in breast cancer survivors. Int J Environ Res Public Health 19 (6), 3278.	No 12-week data. Only 6-week data.
Schröder, M.L., Stöckigt, B., Binting, S., Tissen-Diabaté, T., Bangemann, N., Goerling, U., Kröz, M., Blohmer, J.U., Ortiz, M., Brinkhaus, B., 2022. Feasibility and possible effects of mindful walking and moderate walking in breast cancer survivors: a randomized controlled pilot study with a nested qualitative study part. Integr Cancer Ther 21, 15347354211066067.	No 12-week data. Only 8- and 16-week data.
Boing, L., Fretta, T.B., Lynch, B.M., Dias, M., Rosa, L.M.D., Baptista, F., Bergmann, A., Fausto, D.Y., Bocchi Martins, J.B., Guimarães, A.C.A., 2023. Mat Pilates and belly dance: effects on patient-reported outcomes among breast cancer survivors receiving hormone therapy and adherence to exercise. Complement Ther Clin Pract 50, 101683.	No 12-week data. Only 16-week data.
Dieli-Conwright, C.M., Courneya, K.S., Demark-Wahnefried, W., Sami, N., Lee, K., Sweeney, F.C., Stewart, C., Buchanan, T.A., Spicer, D., Tripathy, D., Bernstein, L., Mortimer, J.E., 2018. Aerobic and resistance exercise improves physical fitness, bone health, and quality of life in overweight and obese breast cancer survivors: a randomized controlled trial. Breast Cancer Res 20 (1), 124.	No 12-week data. Only 16-week data. Enrolled over-weight patients only.
Inbaraj, G., Sathyaprabha, T.N., Udupa, K., Ram, A., Patil, S., Rajeswaran, J., Nandakumar, K.K., Belur, S., Singh, A.D., Prathyusha, P.V., Bayari, S.K., Raghavendra, R.M., 2022. Impact of integrated yoga therapy on cognitive impairment and cardiac dysfunction in relation to quality of life in breast cancer patients undergoing chemotherapy: Study protocol for a two-arm randomized controlled trial. Front Oncol 12, 955184.	No 12-week data. Only 16-week data. Also a during-treatment design, not an after-treatment study.
Ammitzbøll, G., Kristina Kjær, T., Johansen, C., Lanng, C., Wreford Andersen, E., Kroman, N., Zerahn, B., Hyldegaard, O., Envold Bidstrup, P., Oksbjerg Dalton, S., 2019. Effect of progressive resistance training on health-related quality of life in the first year after breast cancer surgery - results from a randomized controlled trial. Acta Oncol 58 (5), 665-672.	No 12-week data. Only 20-week and 12-month data.
Gal, R., Monninkhof, E.M., van Gils, C.H., Groenwold, R.H.H., Elias, S.G., van den Bongard, D., Peeters, P.H.M., Verkooijen, H.M., May, A.M., 2021. Effects of exercise in breast cancer patients: implications of the trials within cohorts (TwiCs) design in the UMBRELLA Fit trial. Breast Cancer Res Treat 190 (1), 89-101.	No 12-week data. Only 6-month data.

Koevoets, E.W., Schagen, S.B., de Ruiter, M.B., Geerlings, M.I., Witlox, L., van der Wall, E., Stuiver, M.M., Sonke, G.S., Velthuis, M.J., Jobsen, J.J., Menke-Pluijmers, M.B.E., Göker, E., van der Pol, C.C., Bos, M., Tick, L.W., van Holsteijn, N.A., van der Palen, J., May, A.M., Monninkhof, E.M., 2022. Effect of physical exercise on cognitive function after chemotherapy in patients with breast cancer: a randomized controlled trial (PAM study). Breast Cancer Res 24 (1), 36.	No 12-week data. Only 6-month data.
van de Wiel, H.J., Stuiver, M.M., May, A.M., van Grinsven, S., Aaronson, N.K., Oldenburg, H.S.A., van der Poel, H.G., Koole, S.N., Retèl, V.P., van Harten, W.H., Groen, W.G., 2021. Effects of and lessons learned from an internet-based physical activity support program (with and without physiotherapist telephone counselling) on physical activity levels of breast and prostate cancer survivors: the PABLO randomized controlled trial. Cancers (Basel) 13 (15), 3665.	No 12-week data. Only 6-month data.
Bruce, J., Mazuquin, B., Canaway, A., Hossain, A., Williamson, E., Mistry, P., Lall, R., Petrou, S., Lamb, S.E., Rees, S., Padfield, E., Vidya, R., Thompson, A.M., 2021. Exercise versus usual care after non-reconstructive breast cancer surgery (UK PROSPER): multicentre randomised controlled trial and economic evaluation. Bmj 375, e066542.	No 12-week data. Only 6- and 12-month data.
Odynets, T., Briskin, Y., Todorova, V., 2019. Effects of different exercise interventions on quality of life in breast cancer patients: a randomized controlled trial. Integr Cancer Ther 18, 1534735419880598.	No 12-week data. Only 6- and 12-month data.
Reeves, M.M., Terranova, C.O., Winkler, E.A.H., McCarthy, N., Hickman, I.J., Ware, R.S., Lawler, S.P., Eakin, E.G., Demark-Wahnefried, W., 2021. Effect of a remotely delivered weight loss intervention in early-stage breast cancer: randomized controlled trial. Nutrients 13 (11), 4091.	No 12-week data. Only 6- and 12-month data.
Vincent, F., Deluche, E., Bonis, J., Leobon, S., Antonini, M.T., Laval, C., Favard, F., Dobbels, E., Lavau-Denes, S., Labrunie, A., Thuillier, F., Venat, L., Tubiana-Mathieu, N., 2020. Home-based physical activity in patients with breast cancer: during and/or after chemotherapy? Impact on cardiorespiratory fitness. A 3-arm randomized controlled trial (APAC). Integr Cancer Ther 19, 1534735420969818.	No 12-week data. Only 6- and 12-month data.
Wang, L.F., Eaglehouse, Y.L., Poppenberg, J.T., Brufsky, J.W., Geramita, E.M., Zhai, S., Davis, K.K., Gibbs, B.B., Metz, J., van Londen, G.J., 2021. Effects of a personal trainer-led exercise intervention on physical activity, physical function, and quality of life of breast cancer survivors. Breast Cancer 28 (3), 737-745.	No 12-week data. Variable follow up periods up to 30 weeks.
Bruce, J., Mazuquin, B., Mistry, P., Rees, S., Canaway, A., Hossain, A., Williamson, E., Padfield, E.J., Lall, R., Richmond, H., Chowdhury, L., Lait, C., Petrou, S., Booth, K., Lamb, S.E., Vidya, R., Thompson, A.M., 2022. Exercise to prevent shoulder problems after breast cancer surgery: the PROSPER RCT. Health Technol Assess 26 (15), 1-124.	No 12-week data. Only 12-month data.
Knoerl, R., Giobbie-Hurder, A., Sannes, T.S., Chagpar, A.B., Dillon, D., Dominici, L.S., Frank, E.S., Golshan, M., McTiernan, A., Rhei, E., Tolaney, S.M., Winer, E.P., Yung, R.L., Irwin, M.L., Ligibel, J.A., 2022. Exploring the impact of exercise and mind-body prehabilitation interventions on physical and psychological outcomes in women undergoing breast cancer surgery. Support Care Cancer 30 (3), 2027-2036.	Not 'after' treatment. A before-treatment study.

An, K.Y., Morielli, A.R., Kang, D.W., Friedenreich, C.M., McKenzie, D.C., Gelmon, K., Mackey, J.R., Reid, R.D., Courneya, K.S., 2020. Effects of exercise dose and type during breast cancer chemotherapy on longer-term patient-reported outcomes and health-related fitness: A randomized controlled trial. Int J Cancer 146 (1), 150-160.	Not 'after' treatment. A during-treatment study.
Bloomquist, K., Adamsen, L., Hayes, S.C., Lillelund, C., Andersen, C., Christensen, K.B., Oturai, P., Ejlertsen, B., Tuxen, M.K., Møller, T., 2019. Heavy-load resistance exercise during chemotherapy in physically inactive breast cancer survivors at risk for lymphedema: a randomized trial. Acta Oncol 58 (12), 1667-1675.	Not 'after' treatment. A during-treatment study.
Cešeiko, R., Eglītis, J., Srebnijs, A., Timofejevs, M., Purmalis, E., Erts, R., Vētra, A., Tomsone, S., 2019. The impact of maximal strength training on quality of life among women with breast cancer undergoing treatment. Exp Oncol 41 (2), 166-172.	Not 'after' treatment. A during-treatment study.
Jacot, W., Arnaud, A., Jarlier, M., Lefeuvre-Plesse, C., Dalivoust, P., Senesse, P., Azzedine, A., Tredan, O., Sadot-Lebouvier, S., Mas, S., Carayol, M., Bleuse, J.P., Gourgou, S., Janiszewski, C., Launay, S., D'Hondt, V., Lauridant, G., Grenier, J., Romieu, G., Ninot, G., Vanlemmens, L., 2020. Brief hospital supervision of exercise and diet during adjuvant breast cancer therapy is not enough to relieve fatigue: a multicenter randomized controlled trial. Nutrients 12 (10), 3081.	Not 'after' treatment. A during-treatment study.
Mavropalias, G., Cormie, P., Peddle-McIntyre, C.J., Galvão, D.A., Taaffe, D.R., Schofield, C., Ray, S., Zissiadis, Y., Newton, R.U., 2023. The effects of home-based exercise therapy for breast cancer-related fatigue induced by radical radiotherapy. Breast Cancer 30 (1), 139-150.	Not 'after' treatment. A during-treatment study.
Rogers, L.Q., Hopkins-Price, P., Vicari, S., Pamenter, R., Courneya, K.S., Markwell, S., Verhulst, S., Hoelzer, K., Naritoku, C., Jones, L., Dunnington, G., Lanzotti, V., Wynstra, J., Shah, L., Edson, B., Graff, A., Lowy, M., 2009. A randomized trial to increase physical activity in breast cancer survivors. Med Sci Sports Exerc 41 (4), 935-946.	Not 'after' treatment. A during-treatment study.
Wonders, K.Y., Schmitz, K., Wise, R., Hale, R., 2022. Cost-Savings Analysis of an Individualized Exercise Oncology Program in Early-Stage Breast Cancer Survivors: A Randomized Clinical Control Trial. JCO Oncol Pract 18 (7), e1170-e1180.	Not 'after' treatment. A during-treatment study.
Wei, X., Yuan, R., Yang, J., Zheng, W., Jin, Y., Wang, M., Jiang, J., Wu, C., Li, K., 2022. Effects of Baduanjin exercise on cognitive function and cancer-related symptoms in women with breast cancer receiving chemotherapy: a randomized controlled trial. Support Care Cancer 30 (7), 6079-6091.	Not 'after' treatment. A during-treatment study.
Sheean, P., Matthews, L., Visotcky, A., Banerjee, A., Moosreiner, A., Kelley, K., Chitambar, C.R., Papanek, P.E., Stolley, M., 2021. Every Day Counts: a randomized pilot lifestyle intervention for women with metastatic breast cancer. Breast Cancer Res Treat 187 (3), 729-741.	Not 'after' treatment. A during-treatment study.
Rogers, L.Q., Hopkins-Price, P., Vicari, S., Markwell, S., Pamenter, R., Courneya, K.S., Hoelzer, K., Naritoku, C., Edson, B., Jones, L., Dunnington, G., Verhulst, S., 2009. Physical activity and health outcomes three months after completing a physical activity behavior change intervention: persistent and delayed effects. Cancer Epidemiol Biomarkers Prev 18 (5), 1410-1418.	Not 'after' treatment. A during-treatment study.

Patel, D.I., Gonzalez, A., Moon, C., Serra, M., Bridges, P.B., Hughes, D., Clarke, G., Kilpela, L., Jiwani, R., Musi, N., 2022. Exercise and creatine supplementation to augment the adaptation of exercise training among breast cancer survivors completing chemotherapy: protocol for an open-label randomized controlled trial (the THRIVE Study). JMIR Res Protoc 11 (4), e26827.	Protocol only
Smith-Turchyn, J., McCowan, M.E., O'Loughlin, E., Fong, A.J., McDonough, M.H., Santa Mina, D., Arbour-Nicitopoulos, K.P., Trinh, L., Jones, J.M., Bender, J.L., Culos-Reed, S.N., Tomasone, J.R., Vani, M.F., Sabiston, C.M., 2021. Connecting breast cancer survivors for exercise: protocol for a two-arm randomized controlled trial. BMC Sports Sci Med Rehabil 13, 128.	Protocol only
Wang, C.C., Geraghty, S., Fox-Harding, C., Wang, C., 2022. Effects of a nurse-led Tai Chi programme on improving quality of life, mental wellbeing, and physical function of women with breast cancer: Protocol for a randomized controlled trial. Womens Health (Lond) 18, 17455057221127813.	Protocol only
Lynch, B.M., Nguyen, N.H., Reeves, M.M., Moore, M.M., Rosenberg, D.E., Wheeler, M.J., Boyle, T., Vallance, J.K., Friedenreich, C.M., English, D.R., 2018. Study design and methods for the ACTIVity And TEchnology (ACTIVATE) trial. Contemp Clin Trials 64, 112-117.	Protocol only
Salchow, J.L., Strunk, M.A., Niels, T., Steck, J., Minto, C.A., Baumann, F.T., 2021. A randomized controlled pilot trial about the influence of Kyusho Jitsu exercise on self-efficacy, fear, depression, and distress of breast cancer patients within follow-up care. Integr Cancer Ther 20, 15347354211037955.	No quality-of-life data
Lin, H.P., Kuo, Y.H., Tai, W.Y., Liu, H.E., 2022. Exercise effects on fatigue in breast cancer survivors after treatments: A systematic review and meta-analysis. Int J Nurs Pract 28 (4), e12989.	No quality-of-life data
Bertoli, J., Bezerra, E.S., Winters-Stone, K.M., Alberto Gobbo, L., Freitas, I.F.J., 2023. Mat Pilates improves lower and upper body strength and flexibility in breast cancer survivors undergoing hormone therapy: a randomized controlled trial (HAPiMat study). Disabil Rehabil 45 (3), 494-503.	No quality-of-life data
Jacquinot, Q., Meneveau, N., Falcoz, A., Bouhaddi, M., Roux, P., Degano, B., Chatot, M., Curtit, E., Mansi, L., Paillard, M.J., Bazan, F., Chaigneau, L., Dobi, E., Meynard, G., Vernerey, D., Pivot, X., Mougin, F., 2022. Cardiotoxicity is mitigated after a supervised exercise program in HER2-positive breast cancer undergoing adjuvant trastuzumab. Front Cardiovasc Med 9, 1000846.	No quality-of-life data
Rogers, L.Q., Courneya, K.S., Carter, S.J., Anton, P.M., Verhulst, S., Vicari, S.K., Robbs, R.S., McAuley, E., 2016. Effects of a multicomponent physical activity behavior change intervention on breast cancer survivor health status outcomes in a randomized controlled trial. Breast Cancer Res Treat 159 (2), 283-291.	Overlapping population with an included trial (Rogers, et al., 2015)
Schleicher, E., McAuley, E., Courneya, K.S., Anton, P., Ehlers, D.K., Phillips, S.M., Oster, R.A., Pekmezi, D., Rogers, L.Q., 2022. Moderators of physical activity and quality of life response to a physical activity intervention for breast cancer survivors. Support Care Cancer 31 (1), 53.	Overlapping population with an included trial (Rogers, et al., 2015)

Holtdirk, F., Mehnert, A., Weiss, M., Mayer, J., Meyer, B., Bröde, P., Claus, M., Watzl, C., 2021. Results of the Optimune trial: a randomized controlled trial evaluating a novel Internet intervention for breast cancer survivors. PLoS One 16 (5), e0251276.	Non-exercise intervention included (cognitive behavioural therapy)
Duijts, S.F., van Beurden, M., Oldenburg, H.S., Hunter, M.S., Kieffer, J.M., Stuiver, M.M., Gerritsma, M.A., Menke-Pluymers, M.B., Plaisier, P.W., Rijna, H., Lopes Cardozo, A.M., Timmers, G., van der Meij, S., van der Veen, H., Bijker, N., de Widt-Levert, L.M., Geenen, M.M., Heuff, G., van Dulken, E.J., Boven, E., Aaronson, N.K., 2012. Efficacy of cognitive behavioral therapy and physical exercise in alleviating treatment-induced menopausal symptoms in patients with breast cancer: results of a randomized, controlled, multicenter trial. J Clin Oncol 30 (33), 4124-4133.	Non-exercise intervention included (cognitive behavioural therapy)
Poier, D., Büssing, A., Rodrigues Recchia, D., Beerenbrock, Y., Reif, M., Nikolaou, A., Zerm, R., Gutenbrunner, C., Kröz, M., 2019. Influence of a multimodal and multimodal-aerobic therapy concept on health-related quality of life in breast cancer survivors. Integr Cancer Ther 18, 1534735418820447.	Non-exercise intervention included (mixed intervention including eurythmy therapy and painting therapy)
Vallance, J.K., Nguyen, N.H., Moore, M.M., Reeves, M.M., Rosenberg, D.E., Boyle, T., Milton, S., Friedenreich, C.M., English, D.R., Lynch, B.M., 2020. Effects of the ACTIVity And TEchnology (ACTIVATE) intervention on health-related quality of life and fatigue outcomes in breast cancer survivors. Psychooncology 29 (1), 204-211.	Non-exercise intervention included (wearable device monitoring)
Chan, D.N.S., Chow, K.M., Anderson, D.J., Porter-Steele, J., Laing, B., Ling, W.M., Lam, C.C.H., Choi, K.C., Chan, C.W.H., So, W.K.W., McCarthy, A.L., 2023. Cultural Adaptation of the Younger Women's Wellness After Cancer Program for Younger Chinese Women With Breast Cancer: A Pilot Randomized Controlled Trial. Cancer Nurs.	Non-exercise intervention included (a comprehensive program beyond exercises)
Naderi, M., Kordestani, H., Sahebi, Z., Khedmati Zare, V., Amani-Shalamzari, S., Kaviani, M., Wiskemann, J., Molanouri Shamsi, M., 2022. Serum and gene expression profile of cytokines following combination of yoga training and vitamin D supplementation in breast cancer survivors: a randomized controlled trial. BMC Womens Health 22 (1), 90.	Non-exercise intervention included (vitamin D)
Cormie, P., Pumpa, K., Galvão, D.A., Turner, E., Spry, N., Saunders, C., Zissiadis, Y., Newton, R.U., 2013. Is it safe and efficacious for women with lymphedema secondary to breast cancer to lift heavy weights during exercise: a randomised controlled trial. J Cancer Surviv 7 (3), 413-424.	Lymphedema patients only
Buchan, J., Janda, M., Box, R., Schmitz, K., Hayes, S., 2016. A randomized trial on the effect of exercise mode on breast cancer-related lymphedema. Med Sci Sports Exerc 48 (10), 1866-1874.	Lymphedema patients only
Zhi, W.I., Baser, R.E., Zhi, L.M., Talukder, D., Li, Q.S., Paul, T., Patterson, C., Piulson, L., Seluzicki, C., Galantino, M.L., Bao, T., 2021. Yoga for cancer survivors with chemotherapy-induced peripheral neuropathy: Health-related quality of life outcomes. Cancer Med 10 (16), 5456-5465.	Included patients other than breast cancer

Ax, A.K., Johansson, B., Lyth, J., Nordin, K., Börjeson, S., 2022. Short- and long-term effect of high versus low-to-moderate intensity exercise to optimise health-related quality of life after oncological treatment-results from the Phys-Can project. Support Care Cancer 30 (7), 5949-5963.	Included patients other than breast cancer
Koch, A.K., Rabsilber, S., Lauche, R., Kümmel, S., Dobos, G., Langhorst, J., Cramer, H., 2017. The effects of yoga and self-esteem on menopausal symptoms and quality of life in breast cancer survivors-A secondary analysis of a randomized controlled trial. Maturitas 105, 95-99.	Incomplete data. Means and standard deviations are not provided.
McNeil, J., Fahim, M., Stone, C.R., O'Reilly, R., Courneya, K.S., Friedenreich, C.M., 2022. Adherence to a lower versus higher intensity physical activity intervention in the Breast Cancer & Physical Activity Level (BC-PAL) Trial. J Cancer Surviv 16 (2), 353-365.	Incomplete data. Means and standard deviations are not provided.
Dong, X., Yi, X., Gao, D., Gao, Z., Huang, S., Chao, M., Chen, W., Ding, M., 2019. The effects of the combined exercise intervention based on internet and social media software (CEIBISMS) on quality of life, muscle strength and cardiorespiratory capacity in Chinese postoperative breast cancer patients:a randomized controlled trial. Health Qual Life Outcomes 17 (1), 109.	Incomplete data. The total score for quality-of-life was not provided.
Strunk, M.A., Zopf, E.M., Steck, J., Hamacher, S., Hallek, M., Baumann, F.T., 2018. Effects of Kyusho Jitsu on physical activity-levels and quality of life in breast cancer patients. In Vivo 32 (4), 819-824.	Incomplete data. The total score for quality-of-life was not provided.

Rogers, L.Q., Courneya, K.S., Anton, P.M., Hopkins-Price, P., Verhulst, S., Vicari, S.K., Robbs, R.S., Mocharnuk, R., McAuley, E., 2015. Effects of the BEAT Cancer physical activity behavior change intervention on physical activity, aerobic fitness, and quality of life in breast cancer survivors: a multicenter randomized controlled trial. Breast Cancer Res Treat 149 (1), 109-119.

Table S4 - Detailed quality assessment of included studies using Cochrane risk of bias 2 tool

First author & Year	Randomization process	Intervention adherence	Missing outcome data	Outcome measurement	Selective reporting	Overall RoB
Milne 2008	L	L[1]	L	L	L	L
Ergun 2013	L	S[2]	L	L	L	S
Baruth 2015	L	L[1]	L	L	L	L
Cramer 2015	L	S[2]	L	L	L	S
Rogers 2015	L	S[2]	L	L	L	S
Stan 2016	L	L[3]	L	L	L	L
Kim 2020	L	S[2]	L	L	L	S
Soriano-Maldonado 2022	L	L[1]	L	L	L	L
Lin 2023	L	S[2]	L	L	L	S

1 The study employed a waitlist control group design, which resulted in a more balanced comparison among different groups.

2 The differences in protocols among various groups may affect adherence and outcome.

3 Both groups were randomized to receive exercise interventions, and the study design utilized a balanced protocol, which minimized the impact on adherence.

H, high risk of bias; L, low risk of bias; S, some risk of bias.

Table S5 - Inconsistency test results of the standardized mean difference in quality of life improvement for breast cancer survivors after 12 weeks of exercise

Comparison	Studies	NMA	Direct	Indirect	Difference	95CIL	95CIU	*p* value
Aerobic : Aerobic+Strength	2	-0.48	-0.18	-1.12	0.94	-0.95	2.83	0.33
Aerobic : Control	3	0.83	0.71	1.33	-0.62	-2.65	1.41	0.55
Aerobic : Strength	0	0.64	-	0.64	-	-	-	-
Aerobic : Yoga	0	0.20	-	0.20	-	-	-	-
Aerobic+Strength : Control	3	1.31	1.42	0.86	0.57	-1.45	2.58	0.58
Aerobic+Strength : Strength	0	1.12	-	1.12	-	-	-	-
Aerobic+Strength : Yoga	0	0.68	-	0.68	-	-	-	-
Strength : Control	1	0.19	-0.30	1.31	-1.61	-4.36	1.14	0.25
Yoga : Control	1	0.63	1.16	-0.45	1.61	-1.14	4.36	0.25
Strength : Yoga	1	-0.44	0.15	-1.46	1.61	-1.14	4.36	0.25

95CIL: lower limit of 95% confidence interval; 95CIU: upper limit of 95% confidence interval; NMA: network meta-analysis

Table S6 - Inconsistency test results for risk difference of dropout rates when applying a 12-week exercise program to improve the quality of life in breast cancer survivors

Comparison	Studies	NMA	Direct	Indirect	Difference	95CIL	95CIU	*p* value
Aerobic : Aerobic+Strength	2	0.01	0.00	0.02	-0.02	-0.12	0.07	0.62
Aerobic : Control	3	0.02	0.03	-0.03	0.06	-0.04	0.16	0.25
Aerobic : Strength	0	0.01	-	0.01	-	-	-	-
Aerobic : Yoga	0	0.04	-	0.04	-	-	-	-
Aerobic+Strength : Control	3	0.01	0.00	0.04	-0.04	-0.14	0.05	0.39
Aerobic+Strength : Strength	0	0.00	-	0.00	-	-	-	-
Aerobic+Strength : Yoga	0	0.03	-	0.03	-	-	-	-
Strength : Control	1	0.01	0.00	0.22	-0.22	-0.54	0.11	0.20
Yoga : Control	1	-0.02	0.00	-0.22	0.22	-0.11	0.54	0.20
Strength : Yoga	1	0.02	0.22	0.00	0.22	-0.11	0.54	0.20

95CIL: lower limit of 95% confidence interval; 95CIU: upper limit of 95% confidence interval; NMA: network meta-analysis

Figure S1

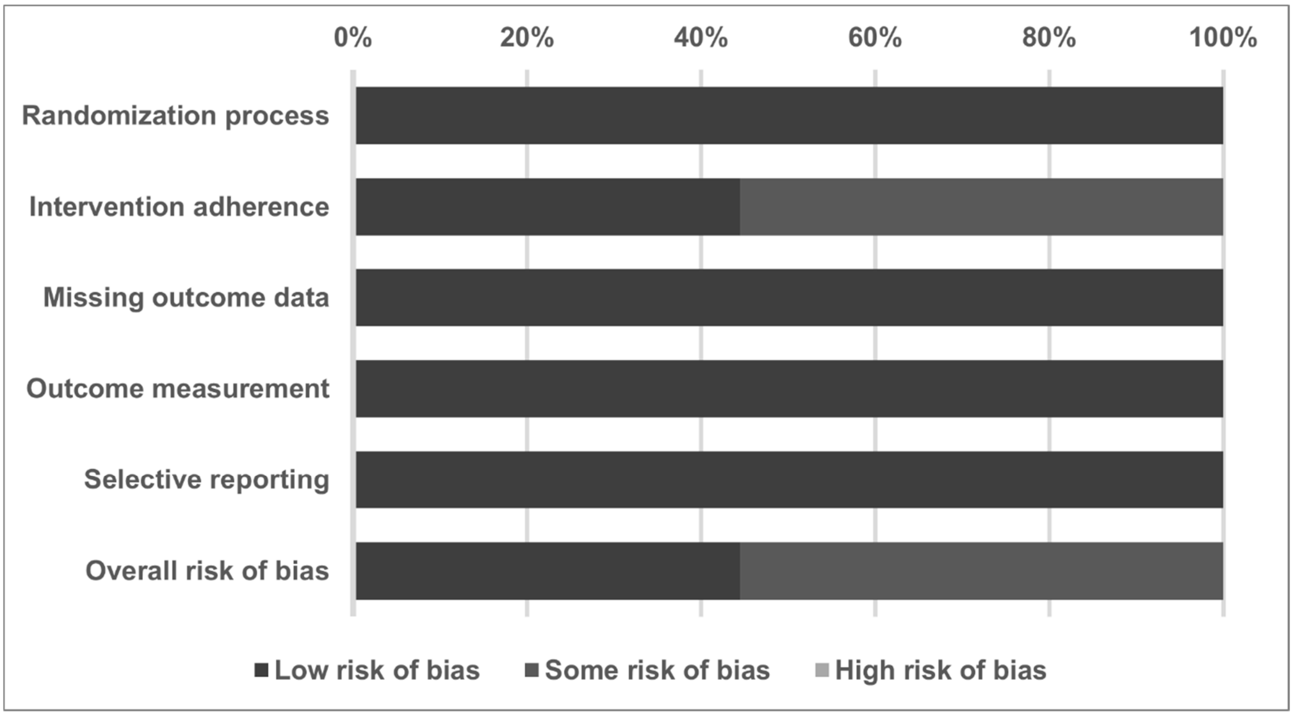

Fig. S1 - Summary of quality assessment for the studies included in the current network meta-analysis using version 2 of the Cochrane risk-of-bias tool for randomized controlled trials. More than half of the studies received some risks in the intervention adherence domain. This is because the interventions in these studies were markedly different, which could affect adherence and outcomes.

Figure S2

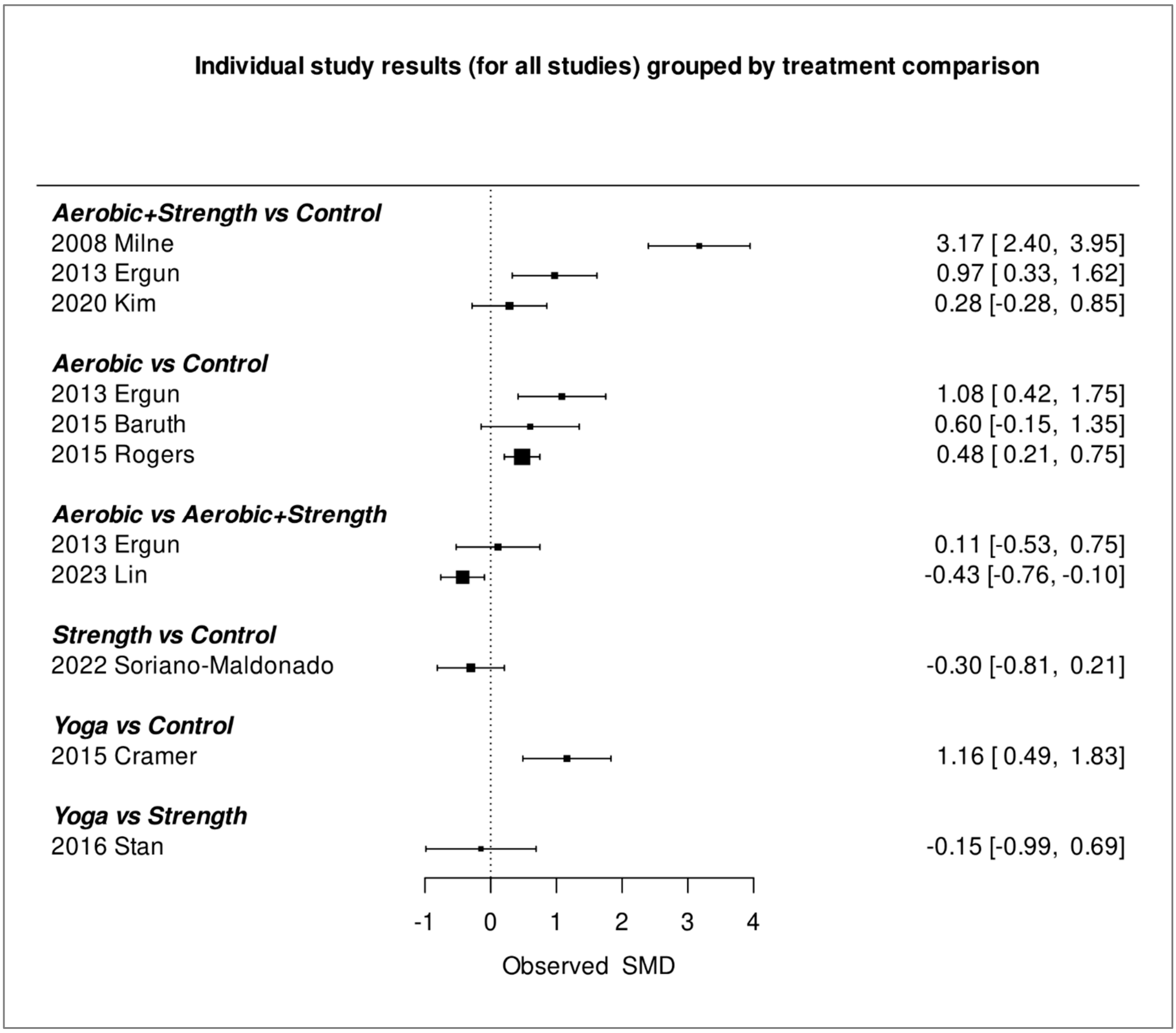

Fig. S2 - The forest plot of pair-wise comparisons for different exercise interventions in breast cancer survivors to improve quality of life, retrieved from the included trials, demonstrates the standardized mean difference (SMD) of quality of life improvement at 12 weeks.

Figure S3

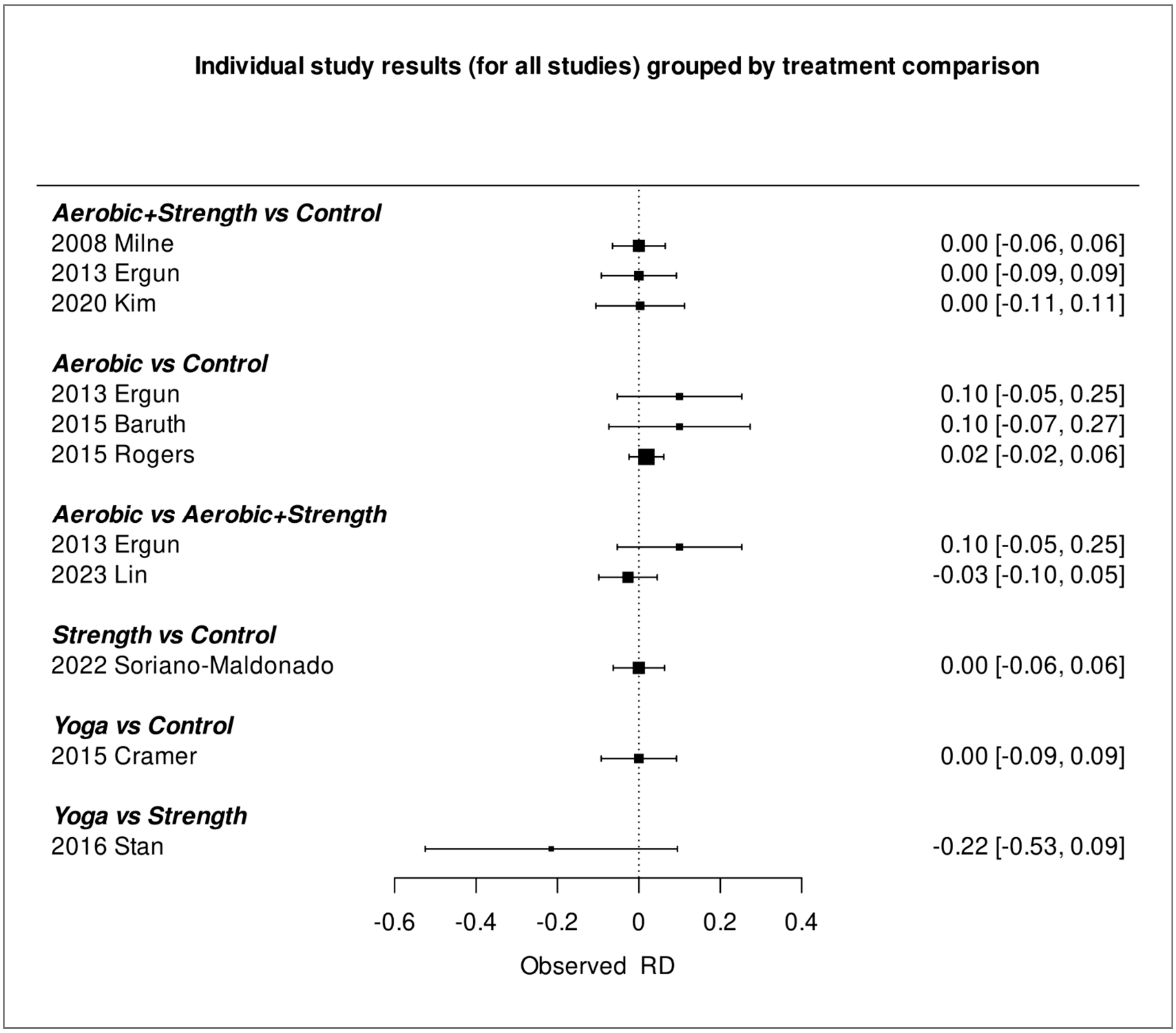

Fig. S3 - The forest plot of pair-wise comparisons for different exercise interventions in breast cancer survivors to improve quality of life, retrieved from the included trials, demonstrates the risk difference (RD) of dropout rates at 12 weeks. None of the comparisons reached statistical significance.

Figure S4

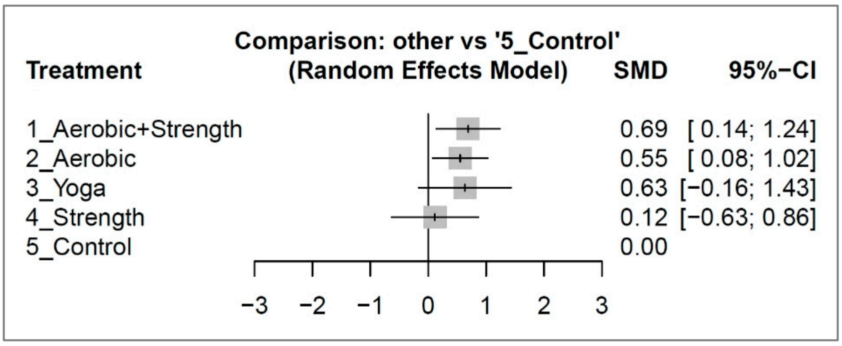

a

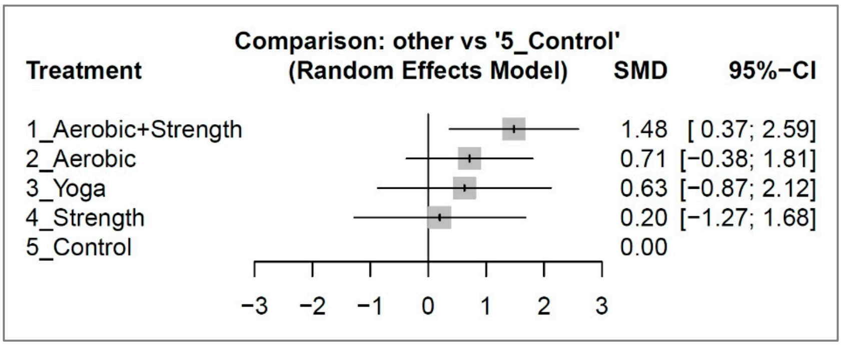

b

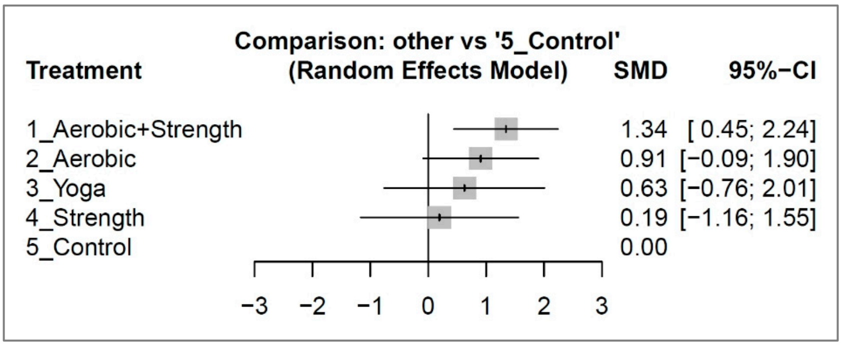

c

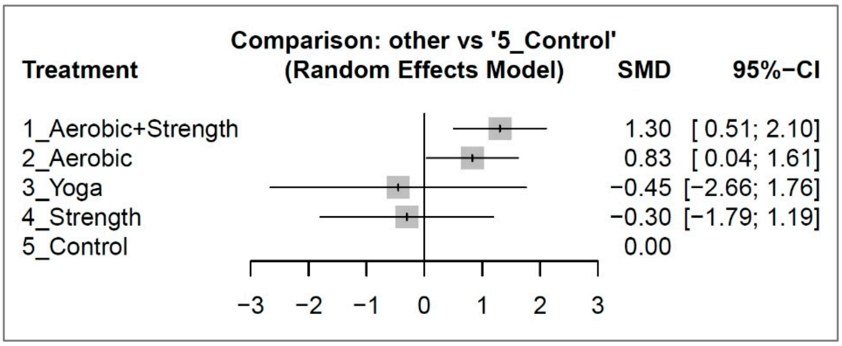

d

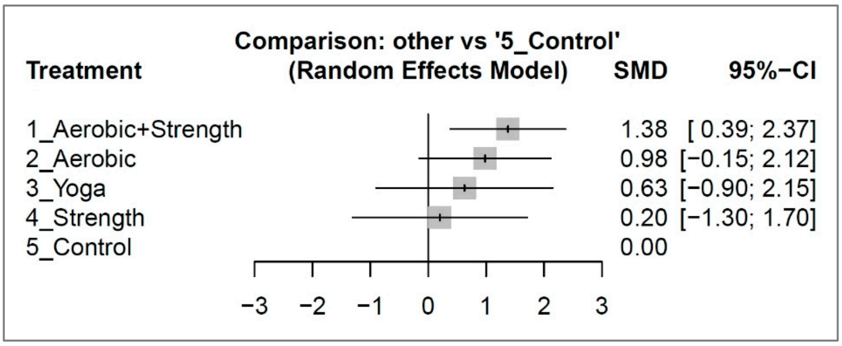

e

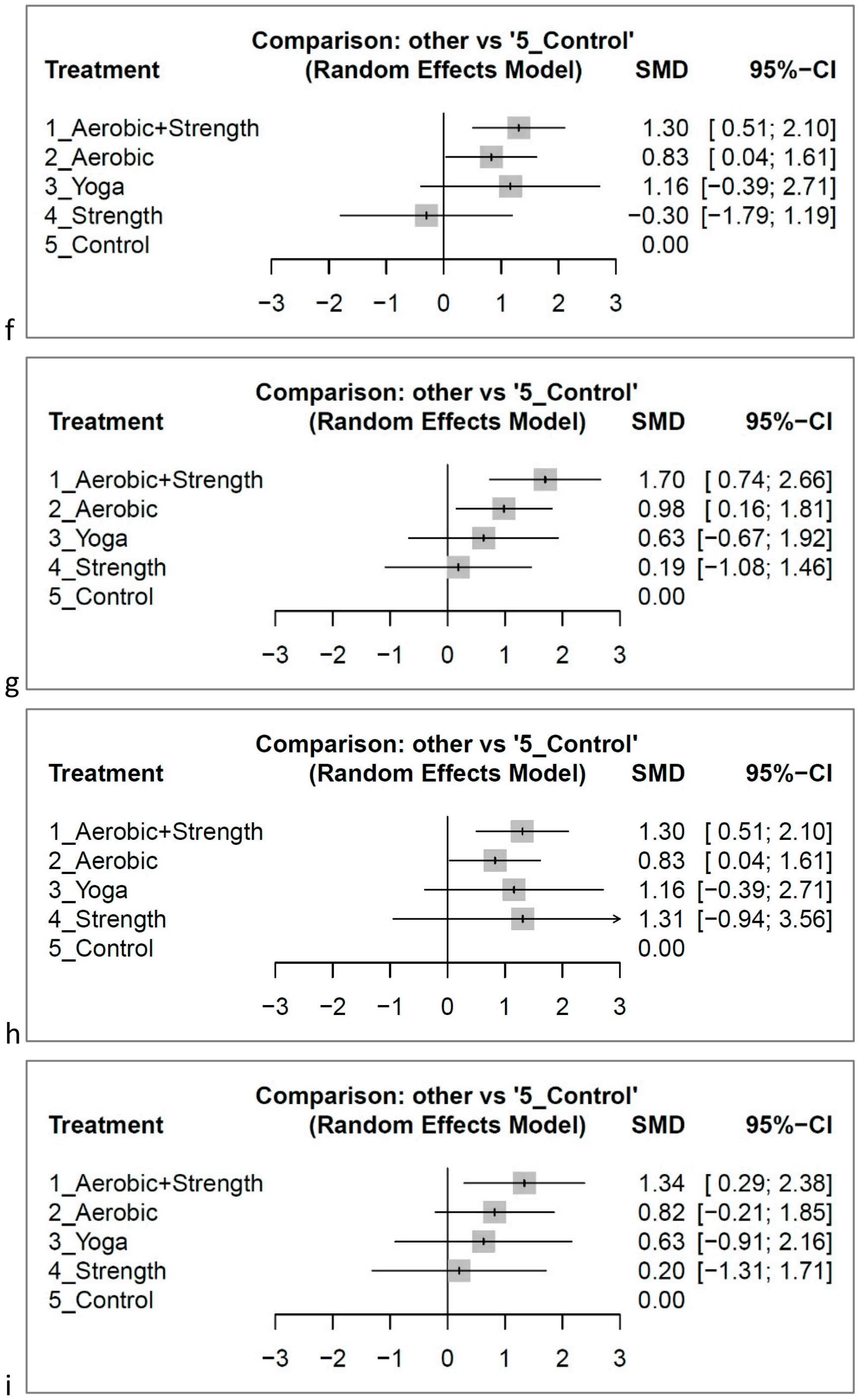

Fig. S4 - Forest plots are presented for sensitivity analysis with the one-study removal method, where each of the nine included studies was sequentially removed based on their year of publication (labeled from a to i). The rankings and clinical implications remained unchanged, indicating that the conclusions of our study are not altered by the inclusion or exclusion of any single study.

Figure S5

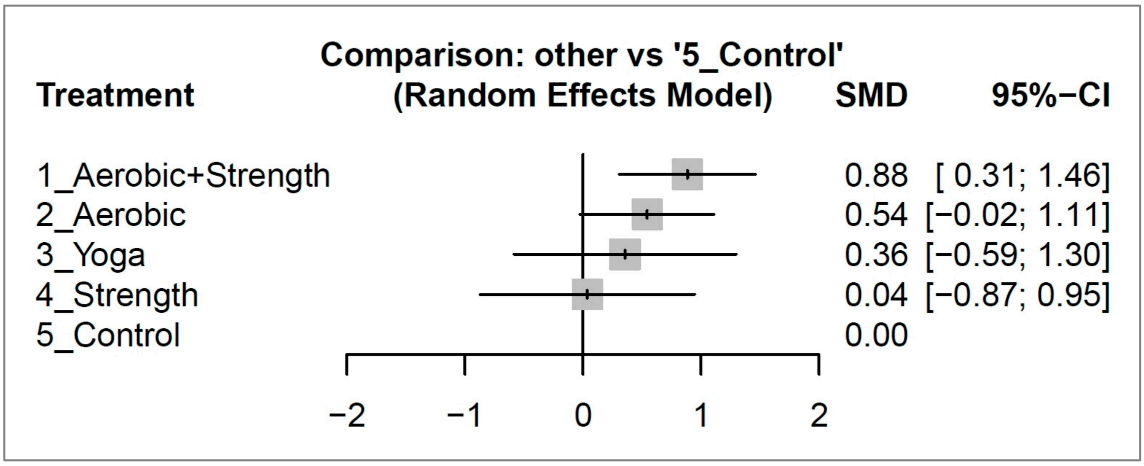

Fig. S5 - Forest plot displaying the improvement in quality of life in breast cancer survivors after receiving different types of exercise interventions, presented as standardized mean differences (SMDs). The pre-post correlation coefficient used in the calculation of data was changed from 0.8 used in Figure 3 to 0.5 in this figure as a sensitivity analysis. The ranking and clinical interpretations remained unchanged compared to Figure 3. This suggests that the conclusions of our study remain unchanged despite different assumptions regarding the coefficient used for transformation.

Figure S6

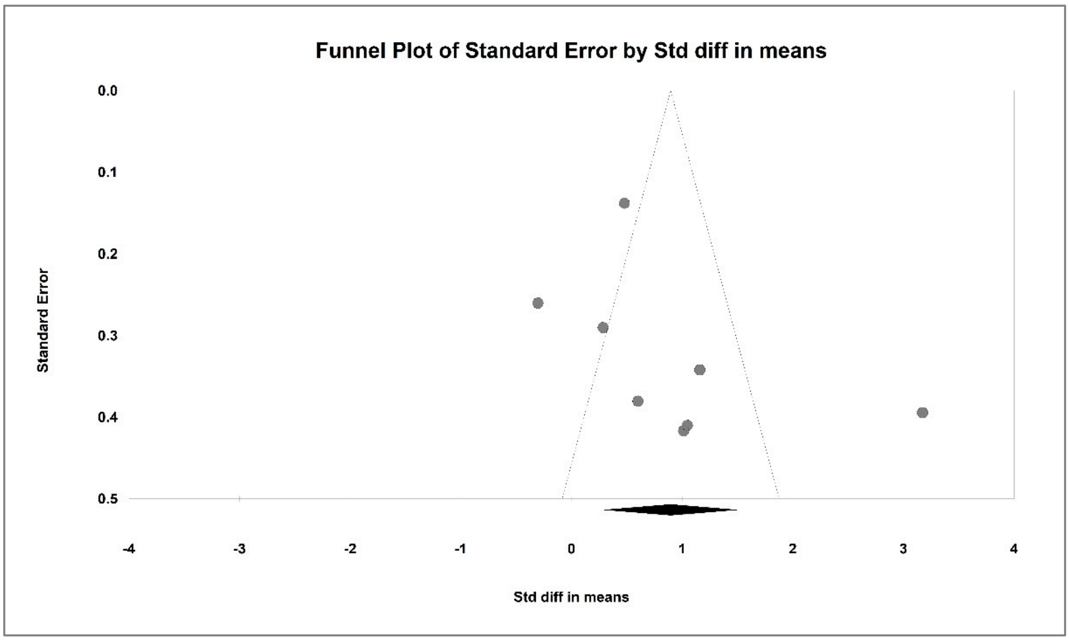

Fig. S6 - Funnel plot of all paired comparisons involving the common comparator, control group. The Egger's test yielded a *p* value of 0.20, indicating no significant publication bias.
